# New insight into the mechanisms protecting bacteria during desiccation

**DOI:** 10.1007/s00294-019-01036-z

**Published:** 2019-09-26

**Authors:** Ewa Laskowska, Dorota Kuczyńska-Wiśnik

**Affiliations:** Faculty of Biology, Department of General and Medical Biochemistry, University of Gdansk, Wita Stwosza 59, 80-308 Gdańsk, Poland

**Keywords:** Advanced glycation end products, Desiccation, Protein acetylation, Protein aggregation

## Abstract

Desiccation is a common stress that bacteria face in the natural environment, and thus, they have developed a variety of protective mechanisms to mitigate the damage caused by water loss. The formation of biofilms and the accumulation of trehalose and sporulation are well-known strategies used by bacteria to survive desiccation. Other mechanisms, including intrinsically disordered proteins and the anti-glycation defence, have been mainly studied in eukaryotic cells, and their role in bacteria remains unclear. We have recently shown that the impairment of trehalose synthesis results in higher glucose availability, leading to the accumulation of acetyl phosphate and enhanced protein acetylation, which in turn stimulates protein aggregation. In the absence of trehalose synthesis, excess glucose may stimulate non-enzymatic glycosylation and the formation of advanced glycation end products (AGEs) bound to proteins. Therefore, we propose that trehalose may prevent protein damage, not only as a chemical chaperone but also as a metabolite that indirectly counteracts detrimental protein acetylation and glycation.

## Introduction

The most common stress that bacteria face in the natural environment is water loss during desiccation. Dehydration results in the condensation of molecules and membrane disruption; reduction of the hydration shell around proteins leads to conformational changes resulting in the loss of enzymatic activity and denaturation. Due to inactivation of the antioxidant mechanisms, free radicals accumulate in the cell causing damage to DNA, proteins and lipids (Lebre et al. 2017). The loss of protein stability and increased molecular crowding favor destructive reactions, including non-enzymatic glycosylation (glycation or the Maillard reaction), leading to irreversible cross-linking and polymerization of proteins and nucleic acids (Boteva and Mironova 2019). Bacteria have elaborated multiple mechanisms that help them to withstand desiccation stress for extended periods. Outbreaks of nosocomial infections are often caused by clinical isolates that can survive on dry surfaces in the hospital environment. Food-borne diseases may be caused by pathogens able to survive the procedures frequently used in the food processing industry, such as desiccation, freeze-drying or hyperosmolarity (Burgess et al. 2016; Lebre et al. 2017). Therefore, the understanding of mechanisms underlying bacterial desiccation tolerance is crucial for human health.

The best characterized strategies involve the formation of biofilms and spores, the accumulation of compatible solutes and synthesis of stress proteins, including molecular chaperones and proteins detoxifying reactive oxygen species. Several excellent reviews have been recently published on these topics (Burgess et al. 2016; Lebre et al. 2017; Esbelin et al. 2018; Vega-Cabrera et al. 2018). Therefore, we focus only on those selected mechanisms that protect proteins during desiccation stress.

### Intrinsically disordered proteins

Late Embryogenesis Abundant (LEA) proteins that accumulate in the late stages of seed maturation were the first identified intrinsically disordered proteins (IDPs) involved in desiccation tolerance (Hincha and Thalhammer 2012). LEA proteins form molecular shields that occupy the space around denatured proteins and inhibit their aggregation (Chakrabortee et al. 2007). Recent studies indicate that IDPs are abundantly present in all proteomes analyzed to date. IDPs can also serve as molecular chaperones, scavengers of reactive oxygen species, hydration buffers or may participate in filament polymerization to maintain higher-order structures in the cell (Boothby and Pielak 2017; Janis et al. 2018). An interesting example of IDPs is the proteins found exclusively in extremotolerant tardigrades. These tardigrade-specific disordered proteins vitrify (form glasses) to reinforce structural integrity of the animal (Boothby et al. 2017). It has been suggested that vitrification protects macromolecules by trapping them within the pores of an amorphous matrix that prevents protein denaturation and aggregation (Boothby and Pielak 2017). Numerous studies have revealed that IDPs participate in a liquid–liquid phase separation (LLPS) of proteins, leading to the formation of dynamic membrane-less organelles, such as Cajal bodies, the nucleolus or stress granules in eukaryotic cells (Shin and Brangwynne 2017; Franzmann and Alberti 2019).

Bacterial proteomes are less disordered than eukaryotic proteomes (Darling and Uversky 2018). Nevertheless, according to the DisProt database [https://beta.disprot.bio.unipd.it (Piovesan et al. 2017)], over 80 *E. coli* IDPs have been identified to date. Moreover, the formation of membrane-less organelles in the cytoplasm and LLPS of bacterial proteins has been reported (Abbondanzieri and Meyer 2019, Monterroso et al. 2019, Al-Husini et al. 2018). It is worth noting that the intracellular environment of prokaryotes is denser than the eukaryotic cytosol, and the bacterial cytoplasm has properties that are characteristic of glass-forming liquids (Parry et al. 2014; Fonseca et al. 2016). Interestingly, it has been demonstrated that cytoplasm fluidity decreases when metabolic activity is inhibited, e.g., during the stationary phase, suggesting that after cytoplasm transition to a solid-like state, bacteria may enter dormancy (Parry et al. 2014). The glass transition of cytoplasm may affect particularly large cellular components, not only by inhibiting their motion but also by stabilizing their structures. A good example is the 100S ribosome which is a dimer of non-translating 70S ribosomes formed constitutively or during the late stationary phase and in response to various stress factors, depending on the bacterial species (Gohara and Yap 2018). Under favorable conditions, these so-called “hibernating ribosomes” disassemble allowing restart of translation. Both ribosome hibernation and the transition of the cytoplasm into a glass-like stage may contribute to the phenomenon of the enhanced ability of bacteria to survive desiccation during stationary phase (Potts 1994).

### Trehalose and Nε-lysine acetylation

A characteristic feature of many anhydrobionts is the accumulation of soluble sugars, including non-reducing disaccharide trehalose (Jain and Roy 2009; Esbelin et al. 2018; Jiang et al. 2018). Trehalose acts as a chemical chaperone, stabilizes denatured proteins and facilitates their refolding in vivo and in vitro (Singer and Lindquist 1998; Diamant et al. 2001; Corradini et al. 2013; Tapia and Koshland 2014). Divergent models have been proposed to explain the protective functions of trehalose. According to the water replacement and exclusion theories, trehalose stabilizes proteins by the displacement of water molecules from their surfaces. The vitrification theory suggests that trehalose forms a viscous glass to shield proteins (Sussich et al. 2001; Crowe et al. 2002; Street et al. 2006; Jain and Roy 2009).

We have shown recently that the synthesis and storage of trehalose in *E. coli* prevent carbon stress, i.e., carbon overflow, which otherwise is manifested by protein acetylation and aggregation (Moruno Algara et al. 2019). The first step of trehalose synthesis is the formation of trehalose-6-phosphate from UDP-glucose and glucose-6-phosphate (Ruhal et al. 2013). In *ΔotsA* cells, unable to synthesize trehalose, the excess glucose-6-phosphate is converted into pyruvate and then into acetyl-CoA and acetyl phosphate (AcP). AcP is engaged in non-enzymatic Nɛ-lysine acetylation of hundreds of *E. coli* proteins (Kuhn et al. 2014; Wolfe 2016). Since AcP is generated in response to carbon overflow, it has been proposed that AcP may be used as a sensor for the nutritional status of the environment, and subsequent acetylation of proteins could limit carbon flux to optimize *E. coli* growth (Christensen et al. 2019). According to this, we observed increased accumulation of AcP and enhanced protein acetylation in *ΔotsA* cells. Since Nε-lysine acetylation neutralizes positively charged lysine-side chains and increases their size and hydrophobicity, acetylation stimulates protein aggregation. (Kuczyńska-Wiśnik et al. 2016; Moruno Algara et al. 2019). These results demonstrated that trehalose may protect proteins against aggregation as a metabolite that indirectly counteracts detrimental acetylation. It should be noted that while some eukaryotic proteins form aggregates after acetylation, others are stabilized in a soluble and active form. The effect of acetylation on aggregation propensity probably depends on the position of lysine-acetylated sites and their functional implications (Cohen et al. 2015; Olzscha et al. 2017; Ferreon et al. 2018).

An alternative mechanism of N-ε lysine acetylation in bacteria involves lysine acetyltransferases (KATs), which catalyze acetylation of specific lysines using acetyl-CoA as the acetyl donor (Christensen et al. 2018). It has been reported that the impairment of KATs-dependent protein acetylation decreases bacterial tolerance to various harmful conditions, including oxidative and high-salt stress (Ma and Wood 2011; Castaño‐Cerezo et al. 2014; Liu et al. 2018). These results suggest that protein acetylation mediated by KATs may be involved in desiccation tolerance; however, further studies are needed to confirm this presumption.

Recent reports indicate that acetylation may affect the structural and functional properties of eukaryotic IDPs (Darling and Uversky 2018). For example, Nε-lysine acetylation of the intrinsically disordered regions in DDX3X, an RNA helicase, inhibited LLPS and the assembly of stress granules (Saito et al. 2019); whereas, hyperacetylation of the microtubule-associated protein Tau disfavored LLPS and inhibited Tau aggregation (Ferreon et al. 2018).

### Anti-glycation defence

**Fig. 1:**
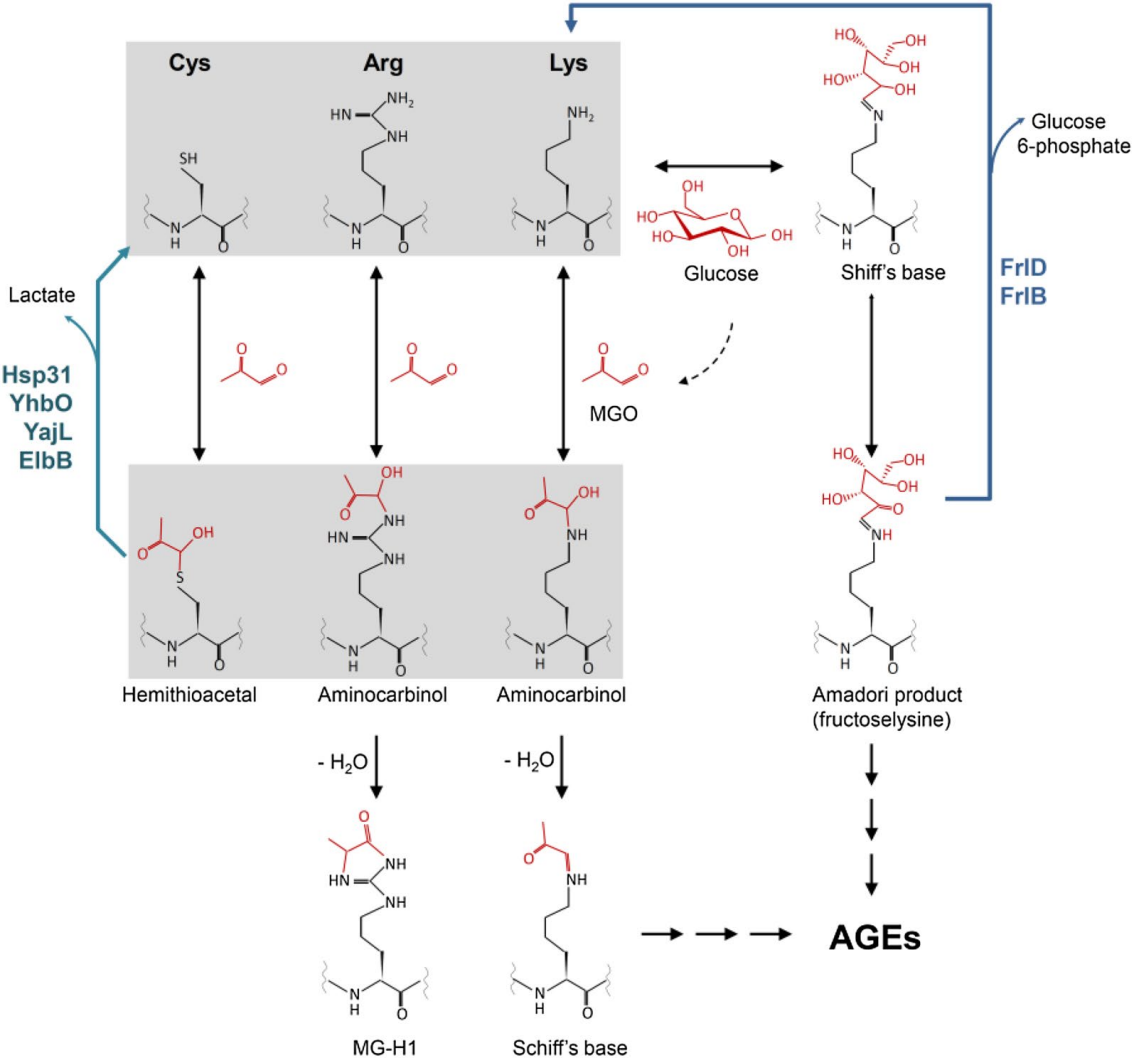
Non-enzymatic glycosylation of proteins. In the initial stage of the Maillard reaction, the aldehyde form of monosaccharides (glucose and fructose) or the glycolytic by-products, such as methylglyoxal (MGO), react spontaneously with thiol and amino groups of proteins. Resulting aminocarbinols (with lysine and arginine) are transformed into Shiff’s bases and next into more stable Amadori products. Advanced glycation end products (AGEs) are formed after additional rearrangements and glycoxidation. *E. coli* enzymes: Hsp31, YhbO, Yaj, ElbB, FrlB and FlrD catalyse deglycation of some adducts. *MGO* methylglyoxal, *MG-H1* hydroimidazolone

Protein glycation (the Maillard reaction) has been studied mainly in eukaryotic systems due to its relation to aging and human diseases. However, there is increasing evidence indicating that protein and DNA glycation also occurs in bacteria despite their short life span (Mironova et al. 2001; Potts et al. 2005; Cohen-Or et al. 2013; Kram and Finkel 2015). Methylglyoxal (MGO) and glyoxal (GO), formed as the by-products of glycolysis or lipid peroxidation (Rabbani and Thornalley 2015), are responsible for up to 60% of glycation damage (Richarme et al. 2018). In the initial stage of the Maillard reaction, the aldehyde form of monosaccharides (glucose and fructose), MGO and GO react spontaneously with proteins (Fig. 1) and nucleic acids. Resulting adducts are sequentially transformed into Shiff’s bases, Amadori products, and finally into advanced glycation end products (AGEs) (Richarme et al. 2018; Boteva and Mironova 2019). AGEs are heterogeneous group of adducts that are prone to aggregation due to intra- and intermolecular crosslinks. Bacteria have developed diverse mechanisms preventing the Maillard reaction. MGO detoxification is catalysed by multiple enzymes including the glyoxylase I/II system, MGO reductase and a group of enzymes converting MGO into acetol. In *E. coli,* four deglycases: Hsp31, YhbO, YalL and ElbB degrade the initial Maillard adducts (Fig. 1). Because these proteins display chaperone activities, they may additionally participate in non-covalent protein repair (Richarme et al. 2018). *E. coli* also produces two enzymes FrlD and FrlB that have been shown to catabolise glycated lysines released upon digestion of food proteins in the human intestine (Wiame et al. 2002). Recent studies indicate that *E. coli* endogenous proteins are substrates for these enzymes: the FrlD kinase phosphorylates fructoselysine (the Amadori product of lysine glycation), whereas FrlB catalyzes the hydrolysis of Nε-fructoselysine 6-phosphate to lysine and glucose 6-phosphate (Fig. 1) (Atanasova et al. 2014). AGEs, the final products of glycation, are degraded by metallo-proteases and secreted by the energy-dependent efflux pump systems (Cohen-Or et al. 2013).

Since both glycation and acetylation may occur on the lysine residue, it seems that these two types of modification compete with each other. Zheng et al. (2019) demonstrated that lysine acetylation protected histones from detrimental glycation in breast cancer cell line. It was also found that Nε lysine acetylation and AGEs formation co-occured in the eye lens molecular chaperones αA- and αB-crystallin (Nahomi et al. 2013). Prior acetylation of αA- and αB-crystallin in vitro before glycation with MGO resulted in significant inhibition of the synthesis of AGEs. Moreover, lysine acetylation prevented loss of chaperone activity of αA- and αB-crystallin caused by glycation.

### Conclusions and perspectives

**Fig. 2:**
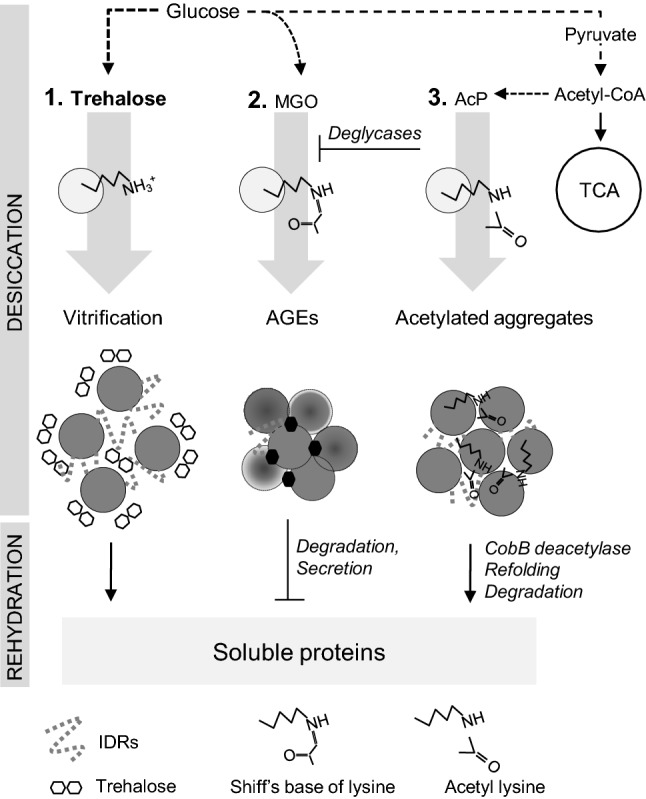
Hypothetical model for the role of trehalose during desiccation. Trehalose protects proteins as a metabolite that indirectly counteracts detrimental protein glycation and acetylation. The lack of trehalose synthesis may result in higher glucose availability which in turn stimulates acetylation and glycation. (1) Trehalose and IDPs allow condensation and transition of soluble proteins into a glass-like state. (2) Protein glycation leads to the formation of AGEs and crosslinked aggregates. (3) AcP-dependent Nε-lysine acetylation neutralizes positively charged lysine side chains and increases hydrophobicity, which in turn, promotes protein aggregation. For clarity, vitrification, glycation and acetylation are shown separately, but all these processes may co-occur in the cell. During rehydration the protein condensates are transformed into a liquid state, the AGEs are degraded and secreted, acetylated and aggregated proteins are solubilized by deacetylation and refolding. *AcP* acetyl phosphate, *AGEs* advanced glycation end products, *IDPs* intrinsically disordered proteins, *MGO* methylglyoxal

The findings presented above suggest that there is a potential interplay between trehalose synthesis and the acetylation and glycation of proteins in bacteria exposed to desiccation stress. We propose that detrimental acetylation and glycation of proteins may be reduced by trehalose synthesis (Fig. 2). This hypothesis is based partly on findings observed in eukaryotic cells, and further studies are needed to verify this model in bacteria. Nevertheless, we have found that in the absence of trehalose synthesis protein, acetylation and aggregation as well as the accumulation of AGEs are enhanced in desiccated *E. coli* cells (Moruno Algara et al. 2019, unpublished results). Both glycation and acetylation may occur on the lysine residue; therefore, it is tempting to speculate that lysine acetylation inhibits the formation of AGEs. This would be beneficial to the cell because acetylation is a reversible modification. In *E. coli,* the deacetylase CobB selectively deacetylates at least some acetyl lysines (Abouelfetouh et al. 2015). Therefore, proteins acetylated upon desiccation can be recovered from the aggregates by deacetylation and refolding; whereas, proteins irreversibly damaged by glycation must be synthesized de novo. In *E. coli*, protein aggregates are deposited at the cell poles due to nucleoid occlusion. In consequence, after cell division aggregates may be inherited only by one of the progeny cells (Winkler et al. 2010). A recent study has demonstrated that the cells inheriting aggregates show increased resistance to subsequent proteotoxic stresses, probably due to co-localisation of molecular chaperones with the aggregates (Mortier et al. 2019). It is plausible that asymmetric segregation of protein aggregates also occurs during rehydration and re-growth of desiccated bacteria. The rapid uptake of water during rehydration may cause cell damage. The question, thus, arises as to which of the two progeny cells, the aggregate-bearing or the aggregate-free cell, is better adapted to the stress imposed by rehydration. The involvement of LPPS in protein aggregation in bacteria is another interesting issue that should be addressed. The aggregates formed in stressed *E. coli* cells contain ribosomal proteins and proteins involved in glycolysis, TCA cycle and other metabolic pathways (Moruno Algara et al. 2019). Some of these proteins possess intrinsically disordered regions; therefore, it is not excluded that the formation of liquid droplets via LLPS precedes aggregation. This hypothesis is consistent with the studies indicating that, with time, proteins concentrated in liquid droplets are converted into aggregates (Patel et al. 2015).

It is worth noting that phase separation of proteins and vitrification of the cytoplasm caused by water loss may induce a dormant state (Parry et al. 2014). Dormant bacteria are difficult to be detected by standard microbiological methods and often acquire antibiotic tolerance. Therefore, the understanding of mechanism underlying tolerance to desiccation stress may help to improve or develop new anti-bacterial strategies.

## References

[CR1] Abbondanzieri EA, Meyer AS (2019). More than just a phase: the search for membraneless organelles in the bacterial cytoplasm. Curr Genet.

[CR2] Abouelfetouh A, Kuhn ML, Hu LI (2015). The *E. coli* sirtuin CobB shows no preference for enzymatic and nonenzymatic lysine acetylation substrate sites. Microbiologyopen.

[CR3] Al-Husini N, Tomares DT, Bitar O (2018). α-Proteobacterial RNA degradosomes assemble Liquid-Liquid Phase-Separated RNP bodies. Mol Cell.

[CR4] Atanasova A, Handzhiyski Y, Sredovska-Bozhinov A (2014). Substrate specificity of the Escherichia coli FRLB amadoriase. Biotechnol Biotechnol Equip.

[CR5] Boothby TC, Pielak GJ (2017). Intrinsically disordered proteins and desiccation tolerance: elucidating functional and mechanistic underpinnings of anhydrobiosis. BioEssays.

[CR6] Boothby TC, Tapia H, Brozena AH (2017). Tardigrades use intrinsically disordered proteins to survive desiccation. Mol Cell.

[CR7] Boteva E, Mironova R (2019). Maillard reaction and aging: can bacteria shed light on the link?. Biotechnol Biotechnol Equip.

[CR8] Burgess CM, Gianotti A, Gruzdev N (2016). The response of foodborne pathogens to osmotic and desiccation stresses in the food chain. Int J Food Microbiol.

[CR9] Castaño-Cerezo S, Bernal V, Post H (2014). Protein acetylation affects acetate metabolism, motility and acid stress response in *Escherichia coli*. Mol Syst Biol.

[CR10] Chakrabortee S, Boschetti C, Walton LJ (2007). Hydrophilic protein associated with desiccation tolerance exhibits broad protein stabilization function. Proc Natl Acad Sci.

[CR11] Christensen DG, Meyer JG, Baumgartner JT (2018). Identification of novel protein lysine acetyltransferases in *Escherichia coli*. MBio.

[CR12] Christensen DG, Baumgartner JT, Xie X (2019). Mechanisms, detection, and relevance of protein acetylation in prokaryotes. MBio.

[CR13] Cohen TJ, Hwang AW, Restrepo CR (2015). An acetylation switch controls TDP-43 function and aggregation propensity. Nat Commun.

[CR14] Cohen-Or I, Katz C, Ron EZ (2013). Metabolism of AGEs–Bacterial AGEs are degraded by metallo-proteases. PLoS One.

[CR15] Corradini D, Strekalova EG, Eugene Stanley H, Gallo P (2013). Microscopic mechanism of protein cryopreservation in an aqueous solution with trehalose. Sci Rep.

[CR16] Crowe JH, Crowe LM, Oliver AE (2002). The trehalose myth revisited: introduction to a symposium on stabilization of cells in the dry state. Cryobiology.

[CR17] Darling AL, Uversky VN (2018). Intrinsic disorder and posttranslational modifications: the darker side of the biological dark matter. Front Genet.

[CR18] Diamant S, Eliahu N, Rosenthal D, Goloubinoff P (2001). Chemical chaperones regulate molecular chaperones in vitro and in cells under combined salt and heat stresses. J Biol Chem.

[CR19] Esbelin J, Santos T, Hébraud M (2018). Desiccation: an environmental and food industry stress that bacteria commonly face. Food Microbiol.

[CR20] Ferreon JC, Jain A, Choi KJ (2018). Acetylation disfavors tau phase separation. Int J Mol Sci.

[CR21] Fonseca F, Meneghel J, Cenard S (2016). Determination of intracellular vitrification temperatures for unicellular micro organisms under conditions relevant for cryopreservation. PLoS One.

[CR22] Franzmann TM, Alberti S (2019). Protein phase separation as a stress survival strategy. Cold Spring Harb Perspect Biol.

[CR23] Gohara DW, Yap MNF (2018). Survival of the drowsiest: the hibernating 100S ribosome in bacterial stress management. Curr Genet.

[CR24] Hincha DK, Thalhammer A (2012). LEA proteins: iDPs with versatile functions in cellular dehydration tolerance. Biochem Soc Trans.

[CR25] Jain NK, Roy I (2009). Effect of trehalose on protein structure. Protein Sci.

[CR26] Janis B, Belott C, Menze MA (2018). Role of intrinsic disorder in animal desiccation tolerance. Proteomics.

[CR27] Jiang H, Liu GL, Chi Z (2018). Genetics of trehalose biosynthesis in desert-derived *Aureobasidium melanogenum* and role of trehalose in the adaptation of the yeast to extreme environments. Curr Genet.

[CR28] Kram KE, Finkel SE (2015). Rich medium composition affects Escherichia coli survival, glycation, and mutation frequency during long-term batch culture. Appl Environ Microbiol.

[CR29] Kuczyńska-Wiśnik D, Moruno-Algara M, Stojowska-Swȩdrzyńska K, Laskowska E (2016). The effect of protein acetylation on the formation and processing of inclusion bodies and endogenous protein aggregates in *Escherichia coli* cells. Microb Cell Fact.

[CR30] Kuhn ML, Zemaitaitis B, Hu LI (2014). Structural, kinetic and proteomic characterization of acetyl phosphate-dependent bacterial protein acetylation. PLoS One.

[CR31] Lebre PH, De Maayer P, Cowan DA (2017). Xerotolerant bacteria: surviving through a dry spell. Nat Rev Microbiol.

[CR32] Liu W, Tan Y, Cao S (2018). Protein acetylation mediated by YfiQ and CobB is involved in the virulence and stress response of Yersinia pestis. Infect Immun.

[CR33] Ma Q, Wood TK (2011). Protein acetylation in prokaryotes increases stress resistance. Biochem Biophys Res Commun.

[CR34] Mironova R, Niwa T, Hayashi H (2001). Evidence for non-enzymatic glycosylation in Escherichia coli. Mol Microbiol.

[CR35] Monterroso B, Zorrilla S, Sobrinos‐Sanguino M (2019). Bacterial FtsZ protein forms phase -separated condensates with its nucleoid‐associated inhibitor SlmA. EMBO Rep.

[CR36] Mortier J, Tadesse W, Govers SK, Aertsen A (2019). Stress-induced protein aggregates shape population heterogeneity in bacteria. Curr Genet.

[CR37] Moruno Algara M, Kuczyńska-Wiśnik D, Dębski J (2019). Trehalose protects *Escherichia coli* against carbon stress manifested by protein acetylation and aggregation. Mol Microbiol.

[CR38] Nahomi RB, Oya-Ito T, Nagaraj RH (2013). The combined effect of acetylation and glycation on the chaperone and anti-apoptotic functions of human α-crystallin. Biochim Biophys Acta - Mol Basis Dis.

[CR39] Olzscha H, Fedorov O, Kessler BM (2017). CBP/p300 bromodomains regulate amyloid-like protein aggregation upon aberrant lysine acetylation. Cell Chem Biol.

[CR40] Parry BR, Surovtsev IV, Cabeen MT (2014). The bacterial cytoplasm has glass-like properties and is fluidized by metabolic activity. Cell.

[CR41] Patel A, Lee HO, Jawerth L (2015). A Liquid-to-solid phase transition of the ALS protein FUS accelerated by disease mutation. Cell.

[CR42] Piovesan D, Tabaro F, Mičetić I (2017). DisProt 7.0: a major update of the database of disordered proteins. Nucleic Acids Res.

[CR43] Potts M (1994). Desiccation tolerance of prokaryotes. Microbiol Rev.

[CR44] Potts M, Slaughter SM, Hunneke F-U (2005). Desiccation tolerance of prokaryotes: application of principles to human cells. Integr Comp Biol.

[CR45] Rabbani N, Thornalley PJ (2015). Dicarbonyl stress in cell and tissue dysfunction contributing to ageing and disease. Biochem Biophys Res Commun.

[CR46] Richarme G, Abdallah J, Mathas N (2018). Further characterization of the Maillard deglycase DJ-1 and its prokaryotic homologs, deglycase 1/Hsp31, deglycase 2/YhbO, and deglycase 3/YajL. Biochem Biophys Res Commun.

[CR47] Ruhal R, Kataria R, Choudhury B (2013). Trends in bacterial trehalose metabolism and significant nodes of metabolic pathway in the direction of trehalose accumulation. Microb Biotechnol.

[CR48] Saito M, Hess D, Eglinger J (2019). Acetylation of intrinsically disordered regions regulates phase separation. Nat Chem Biol.

[CR49] Shin Y, Brangwynne CP (2017). Liquid phase condensation in cell physiology and disease. Science.

[CR50] Singer MA, Lindquist S (1998). Multiple effects of trehalose on protein folding in vitro and in vivo. Mol Cell.

[CR51] Street TO, Bolen DW, Rose GD (2006). A molecular mechanism for osmolyte-induced protein stability. Proc Natl Acad Sci.

[CR52] Sussich F, Skopec C, Brady J, Cesàro A (2001). Reversible dehydration of trehalose and anhydrobiosis: from solution state to an exotic crystal?. Carbohydr Res.

[CR53] Tapia H, Koshland DE (2014). Trehalose is a versatile and long-lived chaperone for desiccation tolerance. Curr Biol.

[CR54] Vega-Cabrera LA, Wood CD, Pardo-López L (2018). Spo0 M: structure and function beyond regulation of sporulation. Curr Genet.

[CR55] Wiame E, Delpierre G, Collard F, Van Schaftingen E (2002). Identification of a pathway for the utilization of the amadori product fructoselysine in *Escherichia coli*. J Biol Chem.

[CR56] Winkler J, Seybert A, Konig L, Prugnaller S, Haselmann U, Sourjik V, Weiss M, Frangakis AS, Mogk A, Bukau B (2010). Quantitative and spatio-temporal features of protein aggregation in *Escherichia coli* and consequences on protein quality control and cellular ageing. EMBO J.

[CR57] Wolfe AJ (2016). Bacterial protein acetylation: new discoveries unanswered questions. Curr Genet.

[CR58] Zheng Q, Omans ND, Leicher R (2019). Reversible histone glycation is associated with disease-related changes in chromatin architecture. Nat Commun.

